# Are birthweight and postnatal weight gain in childhood associated with blood pressure in early adolescence? Results from a Ugandan birth cohort

**DOI:** 10.1093/ije/dyy118

**Published:** 2018-07-03

**Authors:** Swaib A Lule, Benigna Namara, Helen Akurut, Lawrence Muhangi, Lawrence Lubyayi, Margaret Nampijja, Florence Akello, Josephine Tumusiime, Judith C Aujo, Gloria Oduru, Liam Smeeth, Alison M Elliott, Emily L Webb

**Affiliations:** 1 Department of Infectious Disease Epidemiology, London School of Hygiene and Tropical Medicine, London, UK; 2 Endemic, Neglected, Emerging and Re-emerging Infections Programme, MRC/UVRI Uganda Research Unit, Entebbe, Uganda; 3 Maternity Department, Entebbe Hospital, Entebbe, Uganda; 4 Department of Paediatrics, Mulago Hospital, Kampala, Uganda; 5 Department of Non-communicable Disease Epidemiology; 6 Department of Clinical Research, London School of Hygiene and Tropical Medicine, London WC1E 7HT, UK

**Keywords:** Birthweight, postnatal weight change, blood pressure, adolescent, Uganda

## Abstract

**Background:**

In Africa, where low birthweight (LBW), malnutrition and high blood pressure (BP) are prevalent, the relationships between birthweight (BW), weight gain and BP later in life remain uncertain. We examined the effects of early life growth on BP among Ugandan adolescents.

**Methods:**

Data were collected prenatally from women and their offspring were followed from birth, with BP measured following standard protocols in early adolescence. Weight-for-age Z-scores (WAZ) were computed using World Health Organization references. Linear regression was used to relate BW, and changes in WAZ between birth and 5 years, to adolescents’ BP, adjusting for confounders.

**Results:**

Among 2345 live offspring, BP was measured in 1119 (47.7%) adolescents, with mean systolic BP 105.9 mmHg and mean diastolic BP 65.2 mmHg. There was little evidence of association between BW and systolic [regression coefficient β = 0.14, 95% confidence interval (CI) (-1.00, 1.27)] or diastolic [β = 0.43, 95% CI (-0.57, 1.43)] BP. Accelerated weight gain between birth and 5 years was associated with increased BP: systolic β = 1.17, 95% CI (0.69, 1.66) and diastolic β = 1.03, 95% CI (0.59, 1.47). Between birth and 6 months of age, effects of accelerated weight gain on adolescent BP were strongest among the LBW (both premature and small-for-gestational-age) children [BW < 2.5 kg: β = 2.64, 95% CI (0.91, 4.37), BW≥2.5 kg: β = 0.58, 95% CI (0.01, 1.14), interaction *P*-value =  0.024].

**Conclusions:**

Findings from this large tropical birth cohort in Uganda suggest that postnatal weight gain rather than BW is important in the developmental programming of BP, with fast-growing LBW children at particular risk. Efforts to control BP should adopt a life course approach.

## Introduction

Several studies from high-income settings have linked growth in early life (fetal and/or early childhood) with blood pressure (BP) later in life; data suggest an inverse relationship with birthweight (BW) (a proxy for fetal growth)^1^ but a positive association with postnatal weight gain (a proxy for growth in childhood).^2^ The risk is greatest in individuals who experience a ‘nutritional mismatch’ (slow uterine growth leading to low BW followed by accelerated postnatal growth).^2^ Size at birth, postnatal weight gain and ‘nutritional mismatch’ could be important in the developmental programming of later BP.

In Africa, where low BW (LBW),^3–6^ malnutrition (early life undernutrition and later obesity)^7–9^ and high BP^10–12^ are prevalent, the relationship between BW or postnatal weight gain and BP later in life remains unclear. Recent systematic reviews showed that mean systolic and diastolic BP varied from 102.0 mmHg to 118.1 mmHg and from 62.0 mmHg to 71.4 mmHg, respectively, in African children and adolescents aged 5–19,^13^ whereas the prevalence of high BP varied between 1% and 25% among African children and adolescents aged 2–19 years.^14^

In high-income settings, LBW is mainly due to prematurity, which can be due to any of a number of factors (and their combination), including obstetric conditions, maternal body mass index (BMI), maternal smoking and alcohol intake,^15^ whereas in developing countries, small-for-gestational-age (SGA) (often resulting from maternal malnutrition and infections such as HIV and malaria, more prevalent in the tropics) accounts for many LBW infants.^6^^,^^16^ Other causes of LBW such as maternal alcohol intake and smoking are less common in Africa.

The relationships between BW, postnatal weight gain and BP in low-income settings may differ from those seen in high-income countries. Interventions in pregnancy such as prophylactic antimalarial drugs and anthelminthic treatment, common in these settings, have been shown to be associated with birthweight. For example, maternal treatment with mebendazole in pregnancy was associated with increased birthweight in Nepal.^17^ In contrast, our earlier work in Uganda found no impact of maternal treatment with praziquantel and or albendazole in pregnancy on birthweight.^5^ However, the impact of such infections and interventions, and of catch-up nutrition in the small or malnourished African infant, on later BP remains unknown and understudied.

Understanding early life determinants of later BP could be vital in the development of interventions that prevent or control high BP before clinical manifestation of subsequent disease(s). We used data from the Entebbe Mother and Baby Study (EMaBS) birth cohort in Uganda, to investigate the relationship between (i) BW and BP in early adolescence, and (ii) accelerated weight gain in childhood and BP in early adolescence.

## Methods

### Study design and population

Adolescents from the EMaBS, a randomized, double-blind, placebo-controlled trial designed to investigate the effects of worms and their treatment in pregnancy and childhood on vaccine responses and infections in the children,^18^ were enrolled into the BP study.

As previously described,^18^ from 2003 to 2005, pregnant women attending antenatal care at Entebbe Hospital and residing in the study area were enrolled and randomized to receive single-dose praziquantel or matching placebo and single-dose albendazole or matching placebo in a 2 x 2 factorial design. Those with evidence of helminth-induced pathology or history of adverse reaction to anthelminthics or abnormal pregnancy, or who had enrolled for an earlier pregnancy, were excluded.^19^

At 15 months, the resulting offspring were randomized to receive quarterly albendazole or matching placebo up to age 5 years.^18^ Demographic, socioeconomic and health information was collected prenatally (from pregnant women) and from birth onwards from the live-born offspring.

### Measurements

Birthweight was measured immediately after birth using scales (Fazzini SRL, Vimodrone, Italy) for those delivered in Entebbe hospital. For offspring delivered elsewhere, BW was recorded as written on the child health card. Weight was measured at 6 weeks and 6 months of age, using CMS weighing equipment (model MP25: Chasmors Ltd, London, UK) and then annually (close to the child’s birthday) using weighing scales (Seca, Hamburg, Germany). Height was measured as recumbent length at age 6 weeks using an adjustable child-length measuring board (Seca, Hamburg, Germany), then annually (from age 1 year) using stadiometers (Seca 213, Hamburg, Germany). BMI was weight in kilograms (kg) divided by height in metres (m) squared.

Children continued under follow-up after the trial intervention ended in 2011. Between 2 May 2014 and 1 June 2016, those attending their visit at ages 10 or 11 years and not presenting with an illness were enrolled in the BP study; 11-year-olds were excluded if they were previously enrolled as 10-year-olds.

Trained nurses measured BP thrice 5 minutes apart, using an appropriate-sized cuff^20^ on the right arm supported at the heart level, with the participant seated upright all the way to the back of the chair, legs uncrossed and feet flat on the floor. Automated Omron (M6, HEM-700) machines, validated every 6 months by the Uganda National Bureau of Standards, were used. Means of the three systolic and diastolic BP were calculated. Blood pressure percentiles were obtained using Center for Disease Control height percentile charts and National Health and Nutrition Examination Survey Working Group on Children and Adolescents BP tables.^20^^,^^21^ Adolescents with mean BP (systolic and/or diastolic) measurements ≥95th percentile for gender, age and height on day 1 had BP measured for up to two extra days. Those sustaining a high BP on day 3 were referred for specialist attention. Non-pharmacological management was recommended to adolescents with BP (systolic and/or diastolic) ≥90th percentile for age, gender and height.

The study was approved by ethics committees of the Uganda Virus Research Institute, the London School of Hygiene and Tropical Medicine and the Uganda National Council for Science and Technology. Written informed consent and assent were obtained.

### Statistical methods

Data were double-entered in Microsoft Access and analysed using Stata 14 (College Station, TX, USA). Characteristics of cohort members enrolled and not enrolled in the BP study were compared using chi-square tests. The study outcomes were systolic and diastolic BP. The mean of the second and third day-1 BP measurements was used for analysis, as these were on average different from and lower than the first day-1 measurements (Supplementary Figure 1, available as Supplementary data at *IJE* online).

Key exposures were BW and postnatal weight gain. Postnatal weight gain was change in weight-for-age Z-score (WAZ) between birth and age 5 years, with shorter growth periods (birth to 6 months, 6 to 12 months, 12 to 24 months and 24 to 60 months) also examined. The 2006 World Health Organization standard references^22^^,^^23^ were used to calculate WAZ, weight-for-height Z-score (WHZ) and BMI-for-age Z-score (BMIZ).

Potential confounders were maternal characteristics including sociodemographic (age, education, area of residence, socioeconomic status), BMI, pregnancy anthelminthic trial interventions, illness and infections (hypertension, HIV, malaria, worms), and child’s characteristics including sex, feeding status, BMI, age, childhood anthelminthic trial intervention, illness and infections (malaria, worms).

Pearson correlation coefficients between BMI at age 10–11 years and anthropometric variables (WAZ, WHZ and BMIZ) at birth, 6 weeks, 6 months and annually from 1 to 5 years were calculated. Linear regression (fitted separately for systolic and diastolic BP) was used to assess the association between each key exposure and adolescent BP. Adolescents’ age and sex were included a priori in all models. Regression models were adjusted for each potential confounder in turn, with those causing an important change in the effect of the exposure of interest on BP retained in the final model. Final models with and without current weight were fitted.

For BW, we assessed whether a linear or non-linear (categorical or quadratic) relationship provided a better fit to the data. Likelihood ratio test (LRT) was used to examine for effect modification by gender, original trial interventions and birth season. Since the timing of wet seasons in this setting is subject to variability, birth months were categorized as either dry or wet depending on whether the total monthly rainfall was below or above the median rainfall for all birth months.

For weight gain, as well as for those confounders identified in the BW exposure analysis, the effect of each growth period was adjusted for earlier postnatal weight gain period(s) and feeding status at 6 weeks. Effect modification by gender or BW (<2.5 kg versus ≥2.5 kg) was assessed using LRT.

Sensitivity analysis assessing the impact of missing values for the main exposures was conducted: missing BW values were replaced with the minimum (1.26 kg) and the maximum (5.50 kg) non-missing value of BW recorded, and final models re-run for both scenarios. Similarly, missing values for WAZ change were replaced with the smallest and largest change in WAZ for the given growth period.

## Results

Of the 2345 adolescents born into the cohort, 107 (4.6%) had died and 1119 (47.7%) were enrolled. Of those enrolled, 583 (52.1%) were males, 1100 (98.3%) were singletons, 65 (7.0%) had BW<2.5 kg, 824 (73.7%) were delivered and weighed in Entebbe hospital, 651 (58.2%) were delivered in the dry season and 36 (2.9%) were HIV-positive (Supplementary Table 1, available as Supplementary data at *IJE* online).

Participants and non-participants in the BP study were similar for most characteristics (Supplementary Table 1, available as Supplementary data at *IJE* online), with the exceptions that enrolled adolescents were less likely to be HIV positive (*P*-value  =  0.049) but more likely to be singletons (*P*-value  =  0.048), and born to more educated (*P*-value < 0.001) and married/cohabiting mothers (*P*-value  =  0.001), who were less likely to have had hookworm infections (*P*-value  =  0.002) at enrolment.

Participants’ median age was 10.2 years [interquartile range (IQR): 10.0 to 10.9], mean BW was 3.2 kg [standard deviation (SD) 0.5], mean systolic BP was 105.9 mmHg (SD 8.2; range: 74.5–142 mmHg) and mean diastolic BP was 65.2 mmHg (SD 7.3;range: 44-96.5 mmHg). On day 1, 117 (10.5%) had pre-hypertension (mean systolic and/or diastolic BP ≥90th but <95th percentile for gender, age and height), and 94 (8.4%) had hypertension (mean systolic and/or diastolic BP ≥95th percentile for gender, age and height) of whom 76 (80.9%) had stage one and 18 (19.1%) had stage two hypertension. Of those due for a day-2 BP assessment, 72 (76.6%) returned; of these 23 (31.9%) had pre-hypertension and 24 (33.0%) had hypertension. Finally, 18 (75.0%) of those due for a day-3 BP measurement returned, among whom six (33.3%) had pre-hypertension and seven (38.9%) had hypertension. Allowing for loss to follow-up between days 1 to 3, the estimated hypertension prevalence was 1.1%.

**Figure 1:**
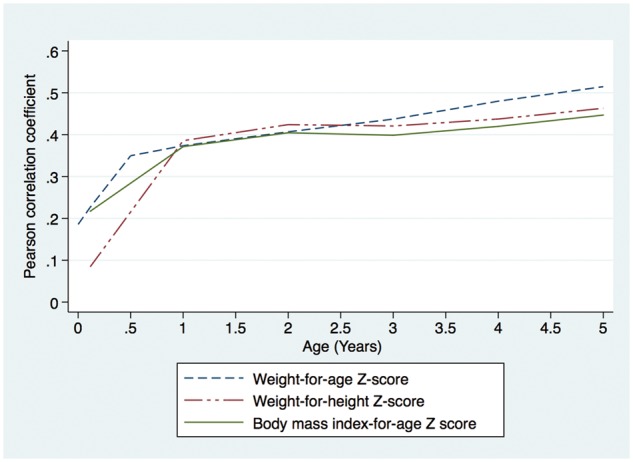
Relationship between anthropometric parameters in childhood and body mass index at the time of blood pressure measurement (10–11 years of age) in adolescents from the Entebbe Mother and Baby Study cohort.

Relationships between anthropometric variables in childhood and BMI at 10–11 years are shown in Figure 1. Correlations between anthropometric variables in childhood and BMI at 10–11 years increased dramatically between birth and 1 year of age, before continuing to increase more gradually. For example, the correlation between WAZ up to and including 6 weeks of age and BMI at age 10–11 years was weak (r < 0.3); the correlation between WAZ at age 0.5–4 years and BMI at age 10–11 years was moderate (0.3≤ r<0.5); and the correlation between WAZ at age 5 years and BMI at age 10–11 years was strong (r≥0.5).

### Effect of birthweight on blood pressure

**Figure 2:**
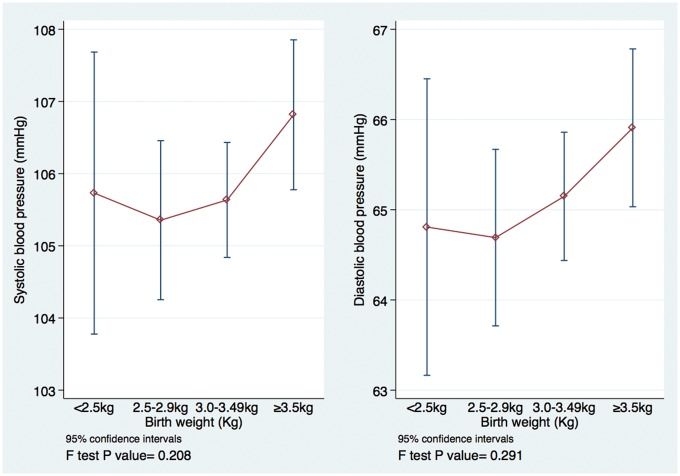
Crude relationship between birthweight and blood pressure among 10- and 11-year olds in the Entebbe Mother and Baby Study.

Crudely, there was a slight suggestion of a U- or J-shaped relationship between BW and BP (Figure 2). Compared with those with BW 3.00–3.49 kg, the difference in adjusted mean systolic BP was +0.30 mmHg, 95% CI (−1.87, +2.47), −0.07 mmHg, 95% CI (−1.50, +1.35) and +0.78 mmHg, 95% CI (−0.56, +2.11) among those with BW <2.5 kg, 2.5–2.99 kg and ≥3.5 kg, respectively (Supplementary Table 3, available as Supplementary data at *IJE* online). However, confidence intervals were wide and treating BW as a categorical variable in the regression model did not improve the fit of the model to the data compared with a linear model (*P*-values for departure from linearity 0.253 and 0.404 for systolic and diastolic BP, respectively). Regardless of whether or not BW was modelled as categorical or linear, there was no evidence for association between BW and BP; for example, linear model regression coefficients were β = 0.14, 95% CI (-1.00, 1.27) for systolic and β = 0.43, 95% CI (-0.57, 1.43) for diastolic BP (Table 1).

**Table 1:** Crude and adjusted effect of birth weight on blood pressure among Entebbe Mother and Baby Study adolescents

Blood pressure	Number	Mean birth weight (Kg)	Mean BP (mmHg)	Crude association	Adjusted association	
				β (95% CI)	β (95% CI)	β (95% CI)^c^
Systolic BP	1119	3.19	105.87	0.73 (−0.33, 1.80)	0.14 (−1.00, 1.27)^a^	−0.91 (−1.99, 0.18)
Diastolic BP	1119	3.19	65.20	0.66 (−0.27, 1.59)	0.43 (−0.57, 1.43)^b^	−0.35 (−1.32, 0.61)

β; linear regression coefficient: mean difference in blood pressure measured in mmHg per 1 kg increase in birth weight, CI: confidence interval.

a Adjusted for maternal factors at enrolment (age, household socioeconomic status, body mass index, asymptomatic malaria, education, parity) and child factors (sex, age, family history of blood pressure).

b Adjusted for maternal factors at enrolment (age, household socioeconomic status, body mass index, asymptomatic malaria, education, parity) and child factors (sex, age, asymptomatic malaria, family history of blood pressure).

c Additionally adjusted for current weight.

**Figure 3:**
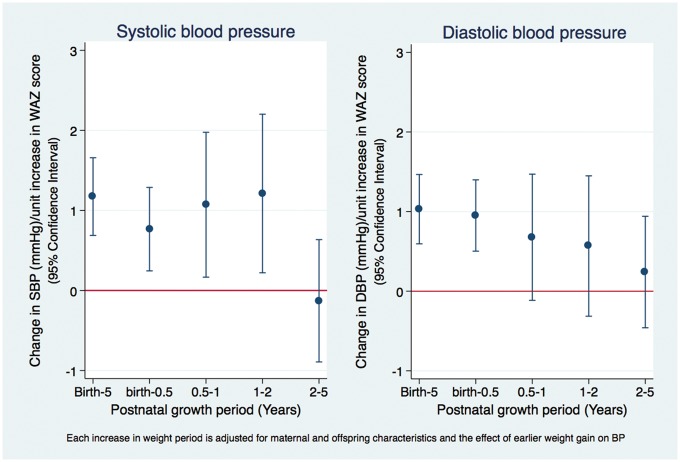
The effect of postnatal weight gain on adolescents’ blood pressure in the Entebbe Mother and Baby Study.

The effect of BW on systolic BP did not differ by sex (interaction *P*-value  =  0.464), birth season (interaction *P*-value  =  0.515) or maternal deworming drugs (praziquantel interaction *P*-value  =  0.230, albendazole interaction *P*-value  =  0.594). Likewise, the effect of BW on diastolic BP did not differ by season of birth, child’s sex or maternal anthelminthic treatment(s). Additionally, adjusting for current weight, the regression coefficients for the effect of BW on systolic BP were -0.91 mmHg, 95% CI (-1.99, 0.18) and -0.35, 95% CI (-1.32, 0.61) for systolic and diastolic BP, respectively. Results from sensitivity analyses were consistent with the main findings (Supplementary Table 2, available as Supplementary data at *IJE* online); for example, the effect of BW on systolic BP varied from a 0.04-mmHg reduction to a 0.07-mmHg increase in systolic BP (compared with the main analysis β point estimate: 0.14 mmHg).

### Effect of weight gain on blood pressure

Crudely, systolic BP was not associated with WAZ at birth [β = 0.34, 95% CI (-0.16, 0.83)] but was strongly associated with WAZ at 6 months [β = 1.14, 95% CI (0.66, 1.61)], 2 year [β = 1.46, 95% CI (1.02, 1.91)] 2 years [β = 1.71, 95% CI (1.23, 2.20)] and 5 years [β = 2.27, 95% CI (1.72, 2.81)]. A similar trend was observed for diastolic BP.

Rapid weight gain between birth and age 5 years was associated with increased BP. Respectively, systolic and diastolic BP increased by 1.17 mmHg and 1.03 mmHg per unit increase in WAZ between birth and 5 years of age (Figure 3). This relationship did not differ by sex or BW (Table 3).

**Table 2:** The association between postnatal weight gain and blood pressure among 10- and 11-year-old adolescents in the Entebbe Mother and Baby Study

Postnatal growth	Number	Crude β (95% CI)	Adjusted β (95% CI)
Systolic blood pressure			
Δ WAZ birth-0.5 year	705	0.71 (0.21, 1.21)	0.77 (0.26, 1.27)^a^
Δ WAZ 0.5-1 year	733	0.76 (−0.04, 1.56)	1.07 (0.16, 1.98)^a^
Δ WAZ 1-2 years	793	0.06 (−0.69, 0.82)	1.21 (0.22, 2.20)^a^
Δ WAZ 2-5 years	802	0.20 (−0.54, 0.94)	−0.13 (−0.89, 0.64)^a^
Δ WAZ Birth-5 years	829	1.17 (0.70, 1.64)	1.17 (0.69, 1.66)^a^
Diastolic blood pressure			
Δ WAZ birth-0.5 year	705	0.88 (0.45, 1.31)	0.95 (0.50, 1.40)^b^
Δ WAZ 0.5-1 year	733	0.45 (−0.25, 1.15)	0.68 (−0.12, 1.47)^b^
Δ WAZ 1-2 years	793	−0.39 (-1.06, 0.27)	0.57 (−0.32, 1.45)^b^
Δ WAZ 2-5 years	802	0.48 (−0.18, 1.15)	0.24 (−0.46, 0.94)^b^
Δ WAZ birth-5 years	829	1.01 (0.59, 1.42)	1.03 (0.59, 1.47)^b^

Δ WAZ: change in weight-for-age-Z score. β: linear regression coefficient: mean difference in blood pressure measured in mmHg per 1 unit increase in ΔWAZ in the specified time interval.

a Adjusted for any earlier postnatal growth period(s), maternal factors at enrolment (age, household socioeconomic status, body mass index, asymptomatic malaria, education, parity) and child factors (sex, age, feeding status at 6 weeks, family history of blood pressure).

b Adjusted for the earlier postnatal growth period, maternal factors at enrolment (age, household socioeconomic status, body mass index, asymptomatic malaria, education, parity) and child factors (sex, age, feeding status at 6 weeks, asymptomatic malaria, family history of blood pressure).

**Table 3:** Effect of postnatal weight gain on blood pressure stratified by birth size and sex among adolescents in the Entebbe Mother and Baby Study

**Postnatal growth period**		**Birthweight <2.5 kg (*n* = 65)**	**Birthweight ≥2.5 kg (*n* = 867)**	**Interaction *P*-value**
		**Adj. β (95% CI)**	**Adj. β (95% CI)**	
**Systolic blood pressure**^a^	
Δ WAZ birth-0.5 year		2.64 (0.91, 4.37)	0.58 (0.01, 1.14)	0.024
Δ WAZ 0.5-1 year		−0.13 (−3.44, 3.19)	1.18 (0.23, 2.13)	0.448
Δ WAZ 1-2 years		2.20 (−0.80, 4.20)	1.16 (0.10, 2.20)	0.504
Δ WAZ 2-5 years		−1.00 (−4.182.18)	0.26 (−0.61, 1.13)	0.444
Δ WAZ birth-5 years		2.41 (0.52, 4.30)	1.30 (0.74, 1.87)	0.268
**Diastolic blood pressure**^b^				
Δ WAZ birth-0.5 year		1.99 (0.49, 3.48)	0.96 (0.47, 1.46)	0.139
Δ WAZ 0.5-1 year		−0.25 (−3.14, 2.65)	0.85 (0.02, 1.68)	0.168
Δ WAZ 1-2 years		0.05 (−2.63, 2.72)	0.82 (−0.12, 1.76)	0.121
Δ WAZ 2-5 years		0.79 (−2.08, 3.66)	0.40 (−0.39, 1.18)	0.793
Δ WAZ birth-5 years		2.08 (0.38, 3.77)	1.20 (0.69, 1.71)	0.323

		**Males (*n* = 583)**	**Females (*n* = 536)**	***P*-value**
		**Adj. β (95% CI)**	**Adj. β (95% CI)**	

**Systolic blood pressure**^a^	
Δ WAZ birth-0.5 year		1.19 (0.50, 1.89)	0.27 (−0.47, 1.01)	0.069
Δ WAZ 0.5-1 year		0.47 (−0.80, 1.74)	1.65 (0.41, 2.89)	0.177
Δ WAZ 1-2 years		1.02 (−0.23, 2.28)	1.47 (−0.02, 2.92)	0.626
Δ WAZ 2-5 years		−0.03 (−1.19, 1.13)	0.40 (−0.81, 1.62)	0.612
Δ WAZ birth-5 years		1.46 (0.80, 2.13)	1.30 (0.51, 2.10)	0.743
**Diastolic blood pressure**^b^				
Δ WAZ birth-0.5 year		1.27 (0.67, 1.88)	0.57 (−0.08, 1.23)	0.116
Δ WAZ 0.5-1 year		0.41 (−0.70, 1.51)	0.95 (−0.15, 2.04)	0.479
Δ WAZ 1-2 years		0.83 (−0.28, 1.95)	0.20 (−1.09, 1.49)	0.437
Δ WAZ 2-5 years		−0.03 (−1.01, 0.94)	0.54 (−0.48, 1.55)	0.424
Δ WAZ birth-5 years		1.103 (0.59, 1.47)	0.83 (0.16, 1.50)	0.438

Δ WAZ: change in weight-for-age-Z score. β: linear regression coefficient: mean difference in blood pressure measured in mmHg per 1 unit increase in ΔWAZ in the specified time interval.

a Each growth period was adjusted for the earlier postnatal growth period, maternal factors at enrolment (age, household socioeconomic status, body mass index, asymptomatic malaria, education, parity) and child’s factors (sex, age, feeding status at 6 weeks, family history of blood pressure).

b Each growth period was adjusted for the earlier postnatal growth period, maternal factors at enrolment (age, household socioeconomic status, body mass index, asymptomatic malaria, education, parity) and child’s factors (sex, age, feeding status at 6 weeks, asymptomatic malaria, family history of blood pressure).

Rapid weight gain during each growth period up to 2 years was independently associated with increased systolic BP, after adjusting for weight gain in the preceding periods. For example, systolic BP increased by 0.77 mmHg, 95% CI (0.26, 1.27) per unit increase in WAZ between birth and age 6 months, by 1.07, 95% CI (0.16, 1.98) between 6 months and 1 year, adjusting for change in WAZ between birth and 6 months, and by 1.21, 95% CI (0.22, 2.20) between 1 and 2 years, adjusting for change in WAZ between birth and 6 months and between 6 months and 1 year (Table 2). Between 2 and 5 years of age, there were no additional effects of increased WAZ on systolic BP among adolescents. For diastolic blood pressure, only rapid weight gain in the first 6 months of life was associated with BP in early adolescence (Table 2).

The effect of accelerated weight gain to age 6 months on systolic BP was stronger among adolescents who were small at birth: β = 2.64, 95% CI (0.91, 4.37) for BW < 2.5 kg and β = 0.58, 95% CI (0.01, 1.14) for BW≥2.5 kg (interaction *P*-value 0.024; Table 3). There was no evidence of effect modification by sex. Results from sensitivity analyses were generally consistent with those from the main analysis (Supplementary Table 4, available as Supplementary data at *IJE* online).

## Discussion

For the first time in mainland East Africa, we described the relationship between BW, postnatal weight gain and BP in adolescents. Birthweight was not associated with BP among early adolescents, but accelerated postnatal weight gain, particularly in the first 2 years of life, was associated with systolic BP, with strongest effects among those born small.

Studies among children and adults have often reported inverse associations between BW and BP, whereas those among adolescents have been inconsistent.^24^^,^^25^ We found no evidence of association, although the relationship between BW and later BP became more inverse on adjusting for current weight, according with previous studies.^26^^,^^27^ We reported results adjusted for current weight, to enable comparison with earlier studies that have done so, although current weight could be considered a mediator rather than a confounder.

Studies from Africa have mainly shown no associations between BW and BP among adolescents (reviewed in Lule *et al.*^13^) Exceptions to this were inverse associations observed among adolescents from Kinshasa, and among boys but not girls from Soweto.^28^^,^^29^ In our study child’s sex, worm treatment in pregnancy, or season of birth did not modify the effect of BW on later BP. As hypothesized, accelerated postnatal weight gain was associated with high BP, with the strongest effects among those born small. This suggests that LBW babies may well be at increased risk of later high BP in this setting, not because of being LBW, but because they are more likely to have rapid early weight gain.

Consistent with literature from high-income countries,^2^^,^^25^^,^^30^ there was a positive association between rapid weight gain and adolescent BP. Few studies from low-middle-income countries have examined the impact of rapid weight gain on later BP. Notably in Seychelles, rapid weight gain was associated with BP in children and adolescents although, contrary to our findings, later rapid weight gain was more strongly associated with later BP than was rapid weight gain in infancy.^31^ Our observation that individuals who were small in early life and experienced accelerated postnatal weight had higher levels of BP accords with findings among Senegalese adults^32^ but differs from results in Chinese children aged 3–6 years.^33^

Findings relating to diastolic BP were consistent with those for systolic BP; thus fetal growth and postnatal growth have similar influences on both systolic and diastolic BP, except that accelerated weight gain up to 2 years of age was associated with systolic BP whereas the effect of rapid weight gain on diastolic BP occurred during the first 6 months.

Strengths of this study included the availability of well-documented prospectively collected data on important covariates including BW, minimizing recall and reporter bias. Exposures and confounders were determined before the BP study was conceptualized and designed. Robust methods were used to measure BP.

Potential limitations include residual confounding from unmeasured confounders, and selection bias resulting from exclusion of pregnancies considered to be abnormal by the midwife (most likely to result in LBW), thus potentially resulting in an underestimation of LBW prevalence or its impact on later BP. Data on prematurity and SGA were not collected, and therefore we were unable to determine the independent effect of each on later BP. Although 187 (16.7%) adolescents were missing BW data, results from sensitivity analyses were similar to the main analysis findings.

Whereas 52% of the adolescents born into this cohort were not enrolled in the BP study, cohort loss to follow-up was below 60%, a level considered to introduce bias if data were missing completely at random or missing at random.^34^ After excluding those who had died by the time of this study, the proportion included in the study was 50%, higher than anticipated (10% loss to follow-up per year was expected).^18^ Furthermore, there were no major differences between cohort members enrolled and not enrolled in the BP study. Our findings are generalizable to this cohort but may not be generalizable to the wider population. Our previous work has suggested that cohort members may, on average, be of somewhat higher socioeconomic status than community residents^35^ and, as a consequence of study participation, they have received free medical care so their health outcomes may be better than in the general population.

Strategies in childhood that prevent excessive weight gain, especially among infants born small, could be important in preventing later high BP. Disease prevention strategies should enhance optimal growth through good maternal and infant nutrition to reduce both LBW and rapid weight gain in early childhood. Future research should seek to evaluate the impact of catch-up nutritional programmes in malnourished infants or infants born small on BP later in life.

In summary, ‘nutritional mismatch’ in early life is critical in the developmental programming of BP among adolescents in developing countries. Strategies that prevent LBW and excessive weight gain in early childhood or reverse their effects on BP could prevent high BP and diseases attributable to high BP.

## Funding

This work was supported by the Wellcome Trust, UK, senior fellowships for A.M.E. [grant numbers 064693, 079110, 95778] with additional support from the UK Medical Research Council and UK Department for International Development (DfID) under the MRC/DfID concordat. S.A.L. was supported by the Commonwealth Scholarship Commission PhD funding at the London School of Hygiene and Tropical Medicine. L.S. was supported in part by the Wellcome Trust, UK [grant number 098504/Z/12/Z]. E.L.W. was supported by the UK Medical Research Council and UK Department for International Development (DfID) under the MRC/DfID concordat [grant number MR/K012126/1].

**Key Messages**

- Among Ugandan adolescents, systolic and diastolic blood pressures were positively associated with accelerated weight gain in early childhood, but not with birthweight.
- The effects of accelerated weight gain in the first years of life on later blood pressure were similar among males and females but differed by size at birth, with the strongest effect among those who were born small.
- Blood pressure control strategies should adopt a life course approach rather than focusing specifically on adults or on the prenatal period.

## Supplementary Material

Supplementary Figure

Supplementary Information
