# Supplementary figures and images for: Are birthweight and postnatal weight gain in childhood associated with blood pressure in early adolescence? Results from a Ugandan birth cohort
Source: Int J Epidemiol. 2018 Jul 3;48(1):148–56. doi: 10.1093/ije/dyy118 (PMC6380421; doi:10.1093/ije/dyy118)

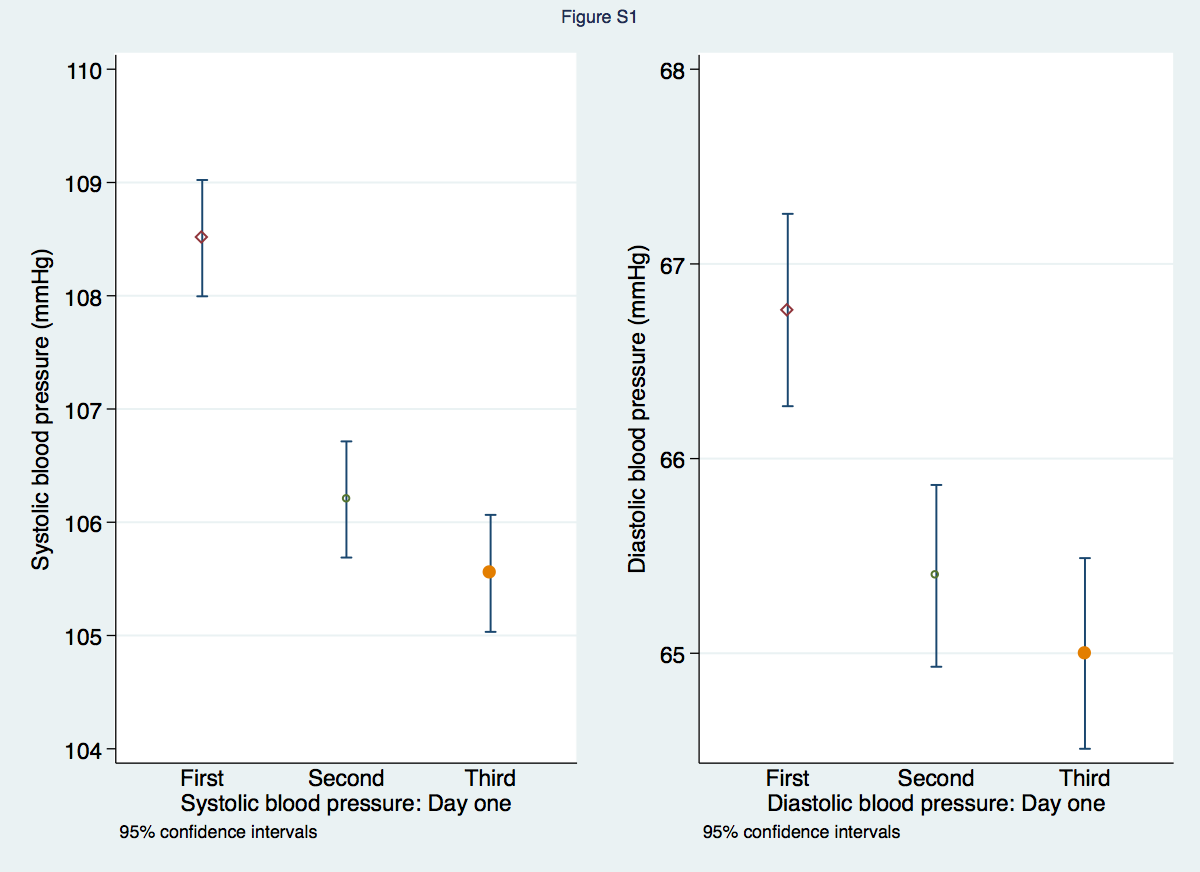

Supplement: Supplementary Figure [file dyy118_supplementary_figure.png]
