# Supplementary material for: Are birthweight and postnatal weight gain in childhood associated with blood pressure in early adolescence? Results from a Ugandan birth cohort
Source: Int J Epidemiol. 2018 Jul 3;48(1):148–56. doi: 10.1093/ije/dyy118 (PMC6380421; doi:10.1093/ije/dyy118)
Supplement: Supplementary Information [file dyy118_supplementary_information.docx]

| **Table S1:** | | | **Characteristics of the Entebbe Mother and Baby Study participants enrolled and not enrolled into the blood pressure study (N=2345)** | | | | |
| --- | --- | --- | --- | --- | --- | --- | --- |
| **Mothers’ characteristics during pregnancy** | | | | **Participated (n=1119)** | | **Did not participate (n=1226)** | |
|  |  |  |  | **Number** | **Percentage/Mean (SD)** | **Number** | **Percentage/Mean (SD)** |
|  | Age (years)^*^ | | | 1119 | 24.4 (5.5) | 1226 | 23.1 (5.1) |
|  | Parity (number of children) ^*^ | | | 1119 | 3.0 (1.8) | 1226 | 2.7 (1.7) |
|  | Body mass index^*^ | | | 1110 | 24.1 (3.4) | 1208 | 24.0 (3.1) |
|  | Household SES | | | 1104 | 3.8 (1.2) | 1197 | 3.5 (1.2) |
|  | Education level | | |  |  |  |  |
|  |  | None | | 28 | 2.5 | 58 | 4.7 |
|  |  | Primary | | 542 | 48.5 | 650 | 53.1 |
|  |  | Senior | | 438 | 39.2 | 433 | 35.4 |
|  |  | Tertiary | | 109 | 9.8 | 83 | 6.8 |
|  | Marital status | | |  |  |  |  |
|  |  | Single | | 116 | 10.4 | 191 | 15.6 |
|  |  | Married/cohabiting | | 967 | 86.5 | 999 | 81.5 |
|  |  | Separated/widowed | | 35 | 3.1 | 36 | 2.9 |
|  | Area or residence | | |  |  |  |  |
|  |  | Urban | | 770 | 69.6 | 831 | 68.9 |
|  |  | Rural | | 336 | 30.4 | 376 | 31.2 |
|  | Infections | | |  |  |  |  |
|  |  | Asymptomatic malaria | | 109 | 9.9 | 139 | 11.6 |
|  |  | Schistosomiasis | | 204 | 18.4 | 217 | 17.7 |
|  |  | Hookworm | | 450 | 40.5 | 575 | 47.0 |
|  |  | Ascaris | | 28 | 2.5 | 26 | 2.1 |
|  |  | Trichuris | | 97 | 8.7 | 109 | 8.9 |
|  |  | Trichostrongylus | | 11 | 1.0 | 11 | 0.8 |
|  | Trial intervention 1 | | |  |  |  |  |
|  |  | Placebo | | 566 | 50.6 | 609 | 49.7 |
|  |  | Albendazole | | 553 | 49.4 | 617 | 50.3 |
|  | Trial intervention 2 | | |  |  |  |  |
|  |  | Placebo | | 564 | 50.4 | 611 | 49.8 |
|  |  | Praziquantel | | 555 | 49.6 | 615 | 50.2 |
| **Participants’ characteristics** | | | |  |  |  |  |
|  | Birthweight (Kg)^*^ | | | 932 | 3.2 (0.5) | 964 | 3.1 (0.5) |
|  | Sex | | |  |  |  |  |
|  |  | Males | | 583 | 52.1 | 628 | 51.3 |
|  | Birth type | | |  |  |  |  |
|  |  | Singleton | | 1100 | 98.3 | 1190 | 97.1 |
|  |  | Multiple | | 19 | 1.7 | 36 | 2.9 |
|  | Feeding at 6 weeks of age | | |  |  |  |  |
|  |  | Exclusive Breastfeeding | | 748 | 67.6 | 724 | 65.3 |
|  |  | Mixed feeding | | 344 | 31.1 | 377 | 34.0 |
|  |  | Weaned | | 14 | 1.3 | 7 | 0.6 |
|  | HIV status | | |  |  |  |  |
|  |  | Unexposed | | 1001 | 89.5 | 1064 | 86.8 |
|  |  | Exposed not infected | | 100 | 8.9 | 126 | 10.3 |
|  |  | Infected | | 18 | 1.6 | 36 | 2.9 |
|  | Birth season | | |  |  |  |  |
|  |  | Wet | | 651 | 58.2 | 683 | 55.9 |
|  |  | Dry | | 468 | 41.8 | 539 | 44.1 |
|  | Place of birth | | |  |  |  |  |
|  |  | Entebbe Hospital | | 824 | 73.7 | 856 | 70.3 |
|  |  | Home | | 120 | 10.7 | 144 | 11.8 |
|  |  | Others | | 174 | 15.6 | 218 | 17.9 |
|  | Mode of delivery | | |  |  |  |  |
|  |  | Normal | | 1005 | 90.0 | 1111 | 91.1 |
|  |  | Caesarean section | | 103 | 9.2 | 94 | 7.7 |
|  |  | Instrumentation | | 9 | 0.8 | 15 | 1.2 |
|  | Trial intervention 3 | | |  |  |  |  |
|  |  | Placebo | | 553 | 50.0 | 453 | 49.8 |
|  |  | Albendazole | | 554 | 50.1 | 456 | 50.2 |
| ^*^ Mean value with standard deviation (SD) presented  Percentages may total to ± 100 due to rounding  Household socioeconomic status (SES) was a composite variable and could take values 1 (low) to 6 (high)  Missing data for (a) mother’s characteristics: body mass index 27; education 4; household SES 44; marital status 1; place of residence 32; asymptomatic malaria 43; schistosomiasis 9; hookworm 9; ascaris 9; trichuris 9; trichostrongylus 9; (b) child’s characteristics: sex 2; birth weight 449; feeding status 131; birth season 4; place of delivery 9; mode of delivery 8; trial intervention 329. | | | | | | | |

| **Table S2:** | | **Crude and adjusted sensitivity analysis for the effect of birth weight on blood pressure among Entebbe Mother and Baby Study adolescents** | | | | | | | | | |
| --- | --- | --- | --- | --- | --- | --- | --- | --- | --- | --- | --- |
| **Blood pressure**  **Sensitivity analysis** | | |  | **Mean birth weight (Kg)** | **Mean BP (mmHg)** | **Crude association** | | **Adjusted association** | | | |
|  |  |  | **Number** |  |  | **β (95% CI)** | **P-value** | **β (95% CI)** | **P-value** | **β (95% CI)^3^** | **P-value** |
| **Lowest birthweight (1.26 kg)** | | | | |  |  |  |  |  |  |  |
|  | Systolic BP | | 1119 | 2.86 | 105.87 | 0.27 (-0.30, 0.84) | 0.353 | -0.04 (-0.80, 0.72)^1^ | 0.913 | -0.53 (-1.26, 0.20) | 0.152 |
|  | Diastolic BP | | 1119 | 2.86 | 65.20 | 0.26 (-0.24, 0.76) | 0.313 | 0.25 (-0.36, 0.86)^2^ | 0.423 | -0.02 (-0.61, 0.57) | 0.955 |
| **Largest birthweight (5.5 kg)** | | | |  |  |  |  |  |  |  |  |
|  | Systolic BP | | 1119 | 3.57 | 105.87 | 0.10 (-0.40, 0.59) | 0.692 | 0.07 (-0.58, 0.71)^1^ | 0.834 | -0.24 (-0.85, 0.37) | 0.438 |
|  | Diastolic BP | | 1119 | 3.57 | 65.20 | 0.08 (-0.36, 0.51) | 0.734 | 0.03 (-0.49, 0.56)^2^ | 0.905 | -0.16 (-0.67, 0.34) | 0.525 |
| β; linear regression coefficient: mean difference in blood pressure measured in mmHg per 1kg increase in birth weight, CI; confidence interval  ^1^ Adjusted for maternal factors at enrolment (age, household socioeconomic status, body mass index, asymptomatic malaria, education, parity) and child factors (sex, age, family history of blood pressure)  ^2^ Adjusted for maternal factors at enrolment (age, household socioeconomic status, body mass index, asymptomatic malaria, education, parity) and child factors (sex, age, asymptomatic malaria, family history of blood pressure)  ^3^ Additionally adjusted for current weight | | | | | | | | | | | |

| **Table S3:** | | **Crude and adjusted relationship between birth weight categories and blood pressure among early adolescents in the Entebbe Mother and Baby Study** | | | | | | | | |
| --- | --- | --- | --- | --- | --- | --- | --- | --- | --- | --- |
| **Birth weight (kg)** | | | **Number (%)** | **Mean (mmHg)** | **Crude β (95% CI)** | **P-value** | **Adjusted β (95% CI)** | **P-value** | **Adjusted β (95% CI)^3^** | **P-value** |
| **a) Systolic blood pressure** | | | | | | | | | | |
|  | <2.50 | | 65 (7.0) | 105.73 | 0.09 (-2.05, 2.24) |  | 0.30 (-1.87, 2.47)^1^ |  | 0.86 (-1.19, 2.90) |  |
|  | 2.50-2.99 | | 207 (22.2) | 105.36 | -0.28 (-1.65, 1.09) |  | -0.07 (-1.50, 1.35)^1^ |  | 0.30 (-1.04, 1.64) |  |
|  | 3.00-3.49 | | 405 (43.5) | 105.65 | 1 |  | 1 |  | 1 |  |
|  | ≥3.50 | | 255 (27.4) | 106.82 | 1.18 (-0.10, 2.46) | 0.208 | 0.78 (-0.56, 2.11)^1^ | 0.666 | -0.06 (-1.33, 1.20) | 0.832 |
| **b) Diastolic blood pressure** | | | |  |  |  |  |  |  |  |
|  | <2.50 | | 65 (7.0) | 64.81 | -0.34 (-2.22, 1.53) |  | 0.17 (-1.64, 1.98)^2^ |  | -0.17 (-2.04, 1.70) |  |
|  | 2.50-2.99 | | 207 (22.2) | 64.69 | -0.46 (-1.66, 0.74) |  | -0.14 (-1.34, 1.06)^2^ |  | -0.38 (-1.62, 0.86) |  |
|  | 3.00-3.49 | | 405 (43.5) | 65.15 | 1 |  | 1 |  | 1 |  |
|  | ≥3.50 | | 255 (27.4) | 65.91 | 0.75 (-0.36, 1.88) | 0.291 | -0.10 (-1.23, 1.02)^2^ | 0.987 | 0.19 (-0.97, 1.35) | 0.879 |
| β; linear regression coefficient: mean difference in BP compared to adolescents weighing 3.00-3.49kg at birth  Percentages may total to ± 100 due to rounding  ^1^ Adjusted for maternal factors at enrolment (age, Household socio-economic status, body mass index, asymptomatic malaria, education, parity,) and child’s factors (sex, age, family history of BP and place of birth)  ^2^ Adjusted for maternal factors at enrolment ((age, household socioeconomic status, body mass index, asymptomatic malaria, education, parity) and child’s factors (sex, age, family history of blood pressure)  ^3^ Additionally adjusted for current weight current | | | | | | | | | | |

| **Table S4** | | **Sensitivity analysis of the association between postnatal weight gain and blood pressure among 10 and 11-year-old adolescents in the Entebbe Mother and Baby Study** | | | | | |
| --- | --- | --- | --- | --- | --- | --- | --- |
| **Lowest change in weight** | | | | | | | |
| **Postnatal growth** | | | **Numbers** | **Crude β (95% CI)** | **P-value** | **Adjusted β (95% CI)** | **P-value** |
| **a) Systolic blood pressure** | | |  |  |  | | |
|  | Δ WAZ Birth-0.5 years | | 1119 | 0.33 (0.11, 0.55) | 0.004 | 0.29 (0.04, 0.54)^1^ | 0.024 |
|  | Δ WAZ 0.5-1 year | | 1119 | 0.46 (0.14, 0.78) | 0.005 | 0.39 (-0.17, 0.95)^1^ | 0.172 |
|  | Δ WAZ 1-2 years | | 1119 | 0.08 (-0.24, 0.40) | 0.622 | 0.12 (-0.45, 0.69)^1^ | 0.679 |
|  | Δ WAZ 2-5 years | | 1119 | -0.11 (-0.42, 0.21) | 0.506 | -0.17 (-0.49, 0.15)^1^ | 0.300 |
|  | **Δ WAZ Birth-5 years** | | **1119** | **0.26 (0.03, 0.50)** | **0.030** | **0.32 (0.05, 0.59)^1^** | **0.019** |
| **b) Diastolic blood pressure** | | |  |  |  | | |
|  | Δ WAZ Birth-0.5 years | | 1119 | 0.38 (0.19, 0.57) | <0.001 | 0.47 (0.25, 0.69)^2^ | <0.001 |
|  | Δ WAZ 0.5-1 year | | 1119 | 0.44 (0.16, 0.72) | 0.002 | 0.33 (-0.16, 0.82)^2^ | 0.184 |
|  | Δ WAZ 1-2 years | | 1119 | 0.01 (-0.28, 0.29) | 0.968 | -0.09 (-0.56, 0.43)^2^ | 0.787 |
|  | Δ WAZ 2-5 years | | 1119 | -0.03 (-0.31, 0.24) | 0.808 | -0.07 (-0.35, 0.22)^2^ | 0.641 |
|  | **Δ WAZ Birth- 5 years** | | **1119** | **0.27 (0.06, 0.48)** | **0.011** | **0.40 (0.16, 0.64)^2^** | **0.001** |
| **Highest change in weight** | | | | | | | |
| **a) Systolic blood pressure** | | |  |  |  |  |  |
|  | Δ WAZ Birth-0.5 years | | 1119 | 0.29 (0.09, 0.49) | 0.005 | 0.25 (0.02, 0.48)^1^ | 0.032 |
|  | Δ WAZ 0.5-1 year | | 1119 | -0.17 (-0.36, 0.03) | 0.093 | 0.20 (-0.18, 0.57)^1^ | 0.300 |
|  | Δ WAZ 1-2 years | | 1119 | -0.05 (-0.33, 0.22) | 0.711 | 0.58 (0.09, 1.07)^1^ | 0.020 |
|  | Δ WAZ 2-5 years | | 1119 | 0.16 (-0.12, 0.44) | 0.267 | 0.12 (-0.17, 0.41)^1^ | 0.410 |
|  | **Δ WAZ Birth -5 years** | | **1119** | **0.26 (0.05, 0.48)** | **0.018** | **0.33 (0.08, 0.58)^1^** | **0.010** |
| **b) Diastolic blood pressure** | | |  |  |  |  |  |
|  | Δ WAZ Birth-0.5 years | | 1119 | -0.05 (-0.26, 0.15) | 0.607 | 0.08 (-0.16, 0.32)^2^ | 0.499 |
|  | Δ WAZ 0.5-1 year | | 1119 | -0.20 (-0.38, -0.03) | 0.019 | 0.04 (-0.28, 0.37)^2^ | 0.791 |
|  | Δ WAZ 1-2 years | | 1119 | -0.12 (-0.37, 0.12) | 0.322 | 0.36 (-0.07, 0.79)^2^ | 0.099 |
|  | Δ WAZ 2-5 years | | 1119 | 0.18 (-0.07, 0.43) | 0.158 | 0.16 (-0.10, 0.41)^2^ | 0.232 |
|  | Δ WAZ **Birth-5 years** | | **1119** | **0.19 (-0.01, 0.38)** | **0.058** | **0.18 (-0.05, 0.40)^2^** | **0.126** |
| Δ WAZ; change in weight-for-age-Z scores, β; linear regression coefficient: mean difference in blood pressure measured in mmHg per 1 unit increase in ΔWAZ  ^1^ Each growth period was adjusted for the earlier postnatal growth period, maternal factors at enrolment (age, household socioeconomic status, body mass index, asymptomatic malaria, education, parity) and child’s factors (sex, age, feeding status at six weeks, family history of blood pressure)  ^2^ Each growth period was adjusted for the earlier postnatal growth period, maternal factors at enrolment (age, household socioeconomic status, body mass index, asymptomatic malaria, education, parity) and child’s factors (sex, age, feeding status at six weeks, asymptomatic malaria, family history of blood pressure) | | | | | | | |
